# Breast cancer histological classification: agreement between the Office for National Statistics and the National Health Service Breast Screening Programme

**DOI:** 10.1186/bcr1352

**Published:** 2005-11-09

**Authors:** Toral Gathani, Diana Bull, Jane Green, Gillian Reeves, Valerie Beral

**Affiliations:** 1 Cancer Epidemiology Unit, Nuffield Department of Clinical Medicine, University of Oxford, Oxford OX3 7LF, UK

## Abstract

**Introduction:**

Epidemiological studies rely on data supplied by central cancer registration sources to be timely, accurate and complete. Validation studies of such data at a national level are limited. Data collected for the Million Women Study was used to compare the level of agreement between the Office for National Statistics (ONS) and the National Health Service Breast Screening Programme (NHSBSP) in the recording of incident screen-detected breast cancer histology between 1996 and 2001.

**Methods:**

1.3 million women aged 50 to 64 years were recruited into the Million Women Study cohort via the NHSBSP. Incident screen-detected breast cancer histologies were notified separately by the ONS and NHSBSP. ICD-10 and ICD-02 ONS codes and NHSBSP histology data were similarly coded to allow for comparison in terms of cancer invasiveness and morphology. The statistical outcome measures are percentage agreement and the kappa statistic.

**Results:**

A total of 5,886 incident screen-detected breast cancers were available for analysis. Of the 5,886 screen-detected cancers reported by the ONS and NHSBSP, 5,684 (96.6%, κ = 0.9) agreed in terms of the degree of invasiveness. Of the 5,458 cancers that had been assigned a specific morphology code, there was exact agreement between the ONS and the NHSBSP in 4,922 cases (90.2%, κ = 0.8).

**Conclusion:**

There is an excellent level of agreement between the ONS and NHSBSP in the recording of the histology of screen-detected breast cancer. From these results it is not possible to comment on which source of data is the more or less accurate, although the differences are very small.

## Introduction

Many epidemiological studies use cancer registration data collected by the Office for National Statistics (ONS) and it is important that the data are reliable. The completeness and accuracy of cancer registration at a national and regional level have been evaluated [1] but no study has specifically examined the agreement in the reporting of breast cancer histology between the ONS and another national database. In this paper, we use data collected in the Million Women Study [2] to compare the agreement in the recording of breast cancer histology between the ONS and the National Health Service Breast Screening Programme (NHSBSP) for incident screen-detected breast cancers.

## Materials and methods

The Million Women Study is a multicentre cohort study involving over one million women across the UK. The methods of the study are described in detail elsewhere [2]. Briefly, women aged between 50 and 64 years and registered within the National Health Service were recruited into the study via the NHSBSP between 1996 and 2001. All women recruited are flagged on the National Health Service Central Register so that incident cancers in the study cohort are notified to the study coordinating centre. In addition, information on screen-detected breast cancer is obtained directly from the NHSBSP. Therefore, for each screen-detected breast cancer, information about the cancer histology has been obtained from two sources, the ONS and NHSBSP. The ONS provides data as ICD-10 site codes and ICD-02 (International Classification of Diseases) histology codes covering both invasive (C50) and non-invasive (D05) breast carcinomas [3]. These codes have been assigned by cancer registry staff at a regional level [4]. The data from the NHSBSP is obtained directly from screening centres. The data are not coded at the centres, and in order to be able to make comparisons between the cancer histology data from each source, the NHSBSP data were classified by Million Women Study staff into one of the 15 categories shown in Table 1. Staff coding the NHSBSP data were unaware of the codes provided by ONS.

This analysis is based on Million Women Study participants who had been diagnosed with a screen detected breast cancer in England between March 1996 and March 2001. Data from the NHSBSP and ONS were compared to see whether they agreed in terms of the degree of cancer invasiveness and with respect to histological classification. In both the ONS and NHSBSP data, if there was notification of both non-invasive and invasive disease in the same woman within a six month time period, the more aggressive tumour recorded was used for comparisons. It was assumed that these notifications related to the same primary cancer. If the NHSBSP recorded more than one invasive histological diagnosis (e.g. invasive ductal cancer and invasive tubular cancer), we allowed any of the diagnoses to agree with the corresponding ONS code for invasive cancer. If the ONS reported more than one breast cancer in the same woman within a six month time period, it was assumed that this was the same primary cancer and only the most aggressive tumour reported was used for coding. When evaluating the level of agreement for histology coding, the analysis was restricted to those neoplasms that had been assigned a specific histology code (Table 1). Analyses were also performed to see how well the data agreed across English regional cancer registries and over time in terms of degree of cancer invasiveness.

Percentage agreement and the kappa statistic are the statistical outcome measures in the study. The kappa statistic is a statistical measure that calculates the agreement between two observations in excess of the amount of agreement that would arise purely by chance. A kappa value (κ) can range from zero to one. A kappa value of 0 indicates that the agreement is due to chance and a value of 1 indicates perfect agreement. Generally a kappa value κ > 0.8 is considered to show very good agreement [5].

All analyses were performed using the statistical package Stata, version 8.0 (Stata Corporation, College Station, TX, USA).

## Results

In total, 5,941 incident screen-detected breast cancers diagnosed between 1996 and 2001 were notified to the Million Women Study by the NHSBSP and ONS. More than one histology code within a six month time period was reported by the ONS in 33 out of 5,941 cases (<1%). In 52 (0.88%) of the cases, histology data from the NHSBSP was incomplete and in 3 (0.05%) of the cases, the ONS histology information was incomplete. These 55 cancers were excluded from the analysis, leaving 5,886 (99%) cases with complete information on histology from both sources available for the comparisons.

### Agreement of invasive versus non-invasive disease

Among the 5,886 screen-detected cancers included in the analysis, the overall prevalence of invasive disease was very similar according to information from both the ONS and NHSBSP, at 80% (4,715/5,886) and 79% (4,621/5,886), respectively (Table 2). In 5,684 (96.6%) cases, there was agreement as to whether the cancer was invasive or non-invasive (κ = 0.90; Table 2). Where there was disagreement, 148 (2.5%) cases were recorded by the ONS as invasive and NHSBSP as non-invasive, whereas only 54 (<1%) cases were recorded by the NHSBSP as invasive and ONS as non-invasive. The disagreement in the recording of invasive and non-invasive disease in these small number of cases was significant (X^2^_1 _= 6.1, p = 0.01).

The level of agreement between invasive and non-invasive disease ranged between 92% and 100% in each calendar year of registration (κ = 0.7 to 0.9: Table 3). There was a significant improvement in the level of agreement over time (X^2^_1 _= 5.2, p = 0.02).

Across the cancer registry regions of England, the level of agreement between invasive and non-invasive disease was between 88% and 98% (κ = 0.9 to 1.0; Table 4).

### Agreement of tumour histology

Of the 5,886 cases shown in Table 2, 377 cases (329 ONS, 66 NHSBSP with 18 cases recorded as 'non-specified invasive histology' by the ONS and NHSBSP) were categorised as 'non-specified invasive histology' and 51 cases (51 ONS, 0 NHSBSP) as 'non-specified non-invasive histology'. This left 5,458 (93%) cases with 1 of 12 specific histology codings (Table 1). Of these 5,458 cases, the ONS and NHSBSP had recorded the same specified histology in 4,922 cases (90.2%, κ = 0.8; Table 5).

For the invasive carcinomas, both sources reported ductal carcinoma as the most common type. Both sources also reported that ductal carcinoma *in situ *(DCIS) is by far the most common type of non-invasive cancer. Tables 6 and 7 show the alternative histology coding assigned to a cancer when the ONS and NHSBSP disagreed over whether the case was invasive ductal carcinoma or DCIS. Six coding categories are shown: invasive ductal carcinoma, 'other specified invasive histology', 'other non-specified invasive histology', DCIS, 'other specified non-invasive histology' and 'other non-specified non-invasive histology'.

Table 6 shows the histology code the NHSBSP reported when the ONS recorded a case as an invasive ductal carcinoma or DCIS. When the ONS recorded invasive ductal carcinoma histology, the NHSBSP did so as well in 93% (3,017/3,228) of cases. Of the remaining 7%, 3% (90/3,228) were coded as 'other specified invasive histology', <1% as other invasive carcinomas with non-specified histology (19/3,228) and 3% (101/3,228) as DCIS. When the ONS recorded histology as DCIS, the NHSBSP did so in 95% (976/1,032) of cases. Of the remaining 6%, 3% (30/1,032) were coded as invasive ductal carcinoma, 2% (20/1,032) as other invasive carcinomas of either specified or non-specified histology and <1% (6/1,032) as non-invasive carcinoma of no specified histology.

Table 7 shows the histology code the ONS reported when the NHSBSP recorded a case as invasive ductal carcinoma or DCIS. When the NHSBSP recorded a case as invasive ductal carcinoma, the ONS assigned the same code in 88% (3,017/3,442) of cases. Of the remaining 12%, 5% (167/3,442) were coded as specified invasive carcinomas with other histologies, 6% (224/3,442) as other invasive carcinomas with non-specified histologies and only 1% (35/3,442) as non-invasive carcinoma. When the NHSBSP recorded a case as DCIS, the ONS did so in 82% (976/1,191) of cases. Of the remaining 18%, 8% (101/1,191) were coded as invasive ductal carcinoma, <1% (8/1,191) as invasive histology of another specified type, 3% (31/1,191) as other invasive histologies of a non-specified type, 3% (30/1,191) as other specified non-invasive histologies and 4% (45/1,191) as other non-invasive histologies of a non-specified type.

## Discussion

The method of cancer registration in England is well documented [4]. The coordinating centre for cancer registration in England is the National Cancer Intelligence Centre, part of the ONS. Until 1993, cancer registration was voluntary. Since January 1993 it has become a mandatory requirement for National Health Service trusts to provide the core items outlined in the cancer registration minimum dataset to the National Cancer Intelligence Centre. Cancer registries obtain free text pathology reports from pathology laboratories and currently data entry in most registries relies on the interpretation of the free text in pathology reports by trained coders. However, the free text in pathology reports is primarily used as a tool for communication between clinicians to enable them to make informed management choices about their patients, not as a tool for cancer registration [6]. It has been shown that guidelines and computerised forms significantly improve the quality of histopathology reporting by pathologists and simplify data entry for coders at the registries [6].

The NHSBSP was set up in 1988 and by the mid 1990s had achieved national coverage. National standards have been set in reference to all aspects of breast screening and these are monitored within a robust national quality assurance network. In addition, the NHSBSP audits its own work through the British Association of Surgical Oncology and other quality assurance groups and standardised pathology reporting is the norm [7]. The guidelines for pathology reporting are detailed and provide a standardised framework through which all pathologists can work [8]. As with the ONS, the NHSBSP extracts data from pathology reports.

The Million Women Study is collecting data on incident screen-detected breast cancers from both the ONS and NHSBSP. Although The Million Women Study has participants in England and Scotland, in this paper we only examine cancer registration in England as the ONS does not cover Scotland. Of the 5,941 incident screen-detected breast cancers diagnosed in study participants between 1996 and 2001, only 55 (1%) cases were excluded from these analyses because of missing data on histology. The histology information from the ONS was extremely complete, with only 3/5,941 (0.05%) cases without such information. The histology information from the NHSBSP was also very complete, with only 52/5,941 (0.88%) cases without such data.

Among women with histology recorded by both the ONS and NHSBSP, the overall level of agreement between the two sources was excellent, with over 95% (κ = 0.9) agreement in terms of degree of invasiveness. Where there was disagreement in regard to the degree of invasiveness, there was a greater tendency for the ONS to report a histology as invasive when the NHSBSP had recorded it as non-invasive, than for the NHSBSP to report a histology as invasive when the ONS recorded a non-invasive histology (2.5% versus <1%). Although the difference in the reporting of invasive and non-invasive histologies was statistically significant, it should be noted that the disagreements are restricted to a very small proportion of the total number of cases (3.5%) and that overall the level of agreement for invasive and non-invasive histologies is excellent.

With regard to the agreement for 12 specified histological types, the level of agreement was still excellent, at around 90% (κ = 0.8), with invasive ductal carcinoma contributing the greatest numbers to the invasive histologies and DCIS contributing the greatest numbers to the non-invasive histologies. When the ONS records an invasive ductal carcinoma, the NHSBSP does so in 93% of cases, and when the ONS records a DCIS, the NHSBSP does so in 94% of cases. However, when the NHSBSP records a diagnosis of invasive ductal carcinoma, the ONS does so in 88% of cases, and when the NHSBSP records a DCIS, the ONS does so in 82% of cases.

In the future, with the planned implementation of the National Health Service National Information Technology (IT) Programme within the next 10 years, cancer registration will be facilitated as information technology practices are standardised across England [9]. The apparent improvement of the levels of agreement over time between the ONS and NHSBSP within the short time period studied was statistically significant; however, it should be noted that the level of agreement was very good in all the years studied. Again, with increasing use of information technology one might expect to see further improvements over time in the future. The minor variation seen between different registries is not unexpected and only one registry had a kappa value of less than 0.8 for agreement between invasive and non-invasive histologies. The local cancer registries are independent and currently differ considerably in their method of data collection, with some being fully automated with all information flowing electronically and others relying on hospital staff to extract data for the registries [4].

The agreement in the recording of breast cancer incidence by the ONS and general practitioner reports has been examined using the General Practice Research Database; the level of agreement between the two sources was found to be high [10]. The Merseyside and Cheshire regional cancer registry has examined all the cancers registered by them in one year and found that the quality of data varied by age of patient, cancer site and area of residence, with breast cancer being no exception [1]. To our knowledge, no existing UK based study has specifically evaluated the agreement of breast cancer histology by the ONS with another national programme.

It is not within the scope of this study to comment on whether the histology data from the ONS are more or less accurate than the histology data from the NHSBSP. We can only comment on what the level of agreement is between them and highlight where disagreements occur. Nevertheless, the results of this study show that the information on breast cancer histology from the ONS is very complete and that there is good agreement with the NHSBSP histology data.

## Conclusion

Information on breast cancer histology from either the ONS or NHSBSP is sufficiently reliable to be used for epidemiological studies, and in particular for long-term prospective studies.

## Abbreviations

DCIS = ductal carcinoma *in situ*; ICD = International Classification of Diseases; NHSBSP = National Health Service Breast Screening Programme; ONS = Office for National Statistics.

## Competing interests

The authors declare that they have no competing interests.

## Authors' contributions

VB conceived of the study. TG drafted the manuscript. DB performed the statistical analysis. All the authors participated in the design and coordination of the study and read and approved the final manuscript.

## Figures and Tables

**Table 1:** Coding categories for breast cancer histologies reported in Million Women Study participants

NHSBSP descriptive data	ONS ICD-02 codes
Invasive ductal carcinoma	8022/3, 8141/3, 8201/3, 8500/3, 8501/3, 8521/3
Invasive lobular carcinoma	8520/3
Invasive tubular carcinoma	8211/3
Invasive mixed carcinoma	8522/3
Invasive mucinous carcinoma	8480/3
Other specified invasive histology	8041/3, 8050/1, 8200/3, 8246/3, 8401/3, 8810/3, 8502/3, 8503/3, 8504/3, 8550/3, 8560/3, 9020/3
Non-specified invasive histology	8530/3, 8140/3, 8000/3, 8001/3, 8003/3, 8010/3
Ductal carcinoma *in situ*	8230/2, 8500/2, 8500/5, 8501/2, 8501/5, 8521/2
Lobular carcinoma *in situ*	8520/2, 8520/5
Ductal + lobular carcinoma *in situ*	8522/2
Pagets	8540/3
Other specified non-invasive histology	8543/3, 8504/2, 8503/2, 8480/5, 8480/2, 8200/2, 8070/2, 8050/5, 8050/2
Non-specified non-invasive histology	8010/2, 8010/5, 8210/2
Unable to code	8000/1, 8520, 8500/6

ICD, International Classification of Diseases; NHSBSP, National Health Service Breast Screening Programme; ONS, Office for National Statistics.

**Table 2:** Overall agreement of invasive and non-invasive histologies in participants of the Million Women Study

	NHSBSP		Total
ONS	Invasive	Non-invasive	

Invasive	4,567	148	4,715
Non-invasive	54	1,117	1,171
Total	4,621	1,265	5,886
			
Percentage agreement			96.6%
Kappa statistic			0.9
Test for heterogeneity			
X^2^_1_			6.1
p			0.01

NHSBSP, National Health Service Breast Screening Programme; ONS, Office for National Statistics.

**Table 3:** Agreement of invasive and non-invasive cancer histologies over time in participants of the Million Women Study

Year	Number of cases	Percentage agreement	κ value
1996	102^a^	0.92	0.73
1997	1,093	0.94	0.84
1998	2,018	0.96	0.89
1999	1,572	0.96	0.89
2000	773	0.97	0.91
2001	315^b^	0.95	0.86

^a^Lower number of cases in 1996 as this is the first year of recruitment into the Million Women Study.

^b^Lower number of cases in 2001 as only cases diagnosed up to the end of March 2001 were included in the analysis. Test for trend: X^2^_1 _= 5.2, p = 0.02.

**Table 4:** Agreement of invasive and non-invasive cancer histologies in the regional cancer registries of England in participants of the Million Women Study

Region	Number of cases^a^	Percentage agreement	κ value
A	1,830	0.96	0.87
B	737	0.97	0.91
C	691	0.97	0.92
D	490	0.95	0.82
E	486	0.94	0.85
F	485	0.96	0.89
G	410	0.98	0.93
H	389	0.97	0.94
I	189	0.97	0.86
J	180	0.88	0.66

^a^Variation in the number of cases is a reflection of the variation in the number of participating breast cancer screening centres within each region.

**Table 5:** Overall agreement where data allow for coding of 12 site specific histologies in participants of the Million Women Study

	NHSBSP												
	Invasive ductal	Invasive lobular	Invasive tubular	Invasive mixed	Other specified invasive	Invasive mucinous	Invasive medullary	Ductal carcinoma *in situ*	Lobular carcinoma *in situ*	Other specified non-invasive	Ductal + lobular carcinoma *in situ*	Pagets	Total

ONS													
Invasive ductal	**3,017**	20	46	14	6	4	0	101	1	1	1	0	3,211
Invasive lobular	32	**515**	9	19	3	3	0	1	4	0	0	0	586
Invasive tubular	39	1	**280**	2	0	0	1	1	0	0	0	0	324
Invasive mixed	68	19	1	**14**	2	1	0	1	0	0	0	0	106
Other specified invasive	21	4	4	0	**23**	0	0	5	0	0	0	0	57
Invasive mucinous	3	0	1	3	1	**42**	1	0	0	0	0	0	51
Invasive medullary	4	0	0	1	0	2	**13**	0	0	0	0	0	20
Ductal carcinoma *in situ*	30	1	0	0	0	0	1	**976**	3	3	8	0	1,022
Lobular carcinoma *in situ*	0	0	0	0	0	0	0	5	**32**	0	5	0	42
Other specified non-invasive	1	0	0	0	0	0	0	18	0	**0**	0	1	20
Ducal + lobular carcinoma *in situ*	0	0	0	0	0	0	0	7	1	0	**10**	0	18
Pagets	0	1	0	0	0	0	0	0	0	0	0	**0**	1
Total	3,215	561	341	53	35	52	16	1,115	41	4	24	1	5,458
Percentage agreement													90%
Kappa statistic						13							0.8

NHSBSP, National Health Service Breast Screening Programme; ONS, Office for National Statistics.

**Table 6:** Number of cases and percentage agreement between Office for National Statistics and National Health Service Breast Screening Programme in relation to invasive ductal carcinoma, ductal carcinoma *in situ *and other invasive and non-invasive histologies in participants of the Million Women Study

	ONS assigned histology code			
	Invasive ductal carcinoma		Ductal carcinoma *in situ*	

NHSBSP histology				
Invasive ductal carcinoma	3,017	93%	30	<1%
Other specified invasive histology	90	3%	4	<1%
Other non-specified invasive histology	19	<1%	16	2%
Ductal carcinoma *in situ*	101	3%	976	95%
Other specified non-invasive histology	1	<1%	6	<1%
Other non-specified non-invasive histology	0	0	0	0
Total	3,228	100%	1,032	100%

NHSBSP, National Health Service Breast Screening Programme; ONS, Office for National Statistics.

**Table 7:** Number of cases and percentage agreement between National Health Service Breast Screening Programme and Office for National Statistics in relation to invasive ductal carcinoma, ductal carcinoma *in situ *and other invasive and non-invasive histologies in participants of the Million Women Study

	NHSBSP assigned histology code			
	Invasive ductal carcinoma		Ductal carcinoma *in situ*	

ONS histology				
Invasive ductal carcinoma	3,017	88%	101	8%
Other specified invasive histology	167	5%	8	<1%
Other non-specified invasive histology	224	6%	31	3%
Ductal carcinoma *in situ*	30	<1%	976	82%
Other specified non-invasive histology	1	<1%	30	3%
Other non-specified non-invasive histology	3	<1%	45	4%
Total	3,442	100%	1,191	100%

NHSBSP, National Health Service Breast Screening Programme; ONS, Office for National Statistics.
